# Spatial Modulation of Primate Inferotemporal Responses by Eye Position

**DOI:** 10.1371/journal.pone.0003492

**Published:** 2008-10-23

**Authors:** Sidney R. Lehky, Xinmiao Peng, Carrie J. McAdams, Anne B. Sereno

**Affiliations:** 1 Computational Neuroscience Laboratory, The Salk Institute, La Jolla, California, United States of America; 2 Department of Neurobiology and Anatomy, University of Texas Houston Health Science Center, Houston, Texas, United States of America; 3 Department of Psychiatry, University of Texas Southwestern Medical Center, Dallas, Texas, United States of America; Lund University, Sweden

## Abstract

**Background:**

A key aspect of representations for object recognition and scene analysis in the ventral visual stream is the spatial frame of reference, be it a viewer-centered, object-centered, or scene-based coordinate system. Coordinate transforms from retinocentric space to other reference frames involve combining neural visual responses with extraretinal postural information.

**Methodology/Principal Findings:**

We examined whether such spatial information is available to anterior inferotemporal (AIT) neurons in the macaque monkey by measuring the effect of eye position on responses to a set of simple 2D shapes. We report, for the first time, a significant eye position effect in over 40% of recorded neurons with small gaze angle shifts from central fixation. Although eye position modulates responses, it does not change shape selectivity.

**Conclusions/Significance:**

These data demonstrate that spatial information is available in AIT for the representation of objects and scenes within a non-retinocentric frame of reference. More generally, the availability of spatial information in AIT calls into questions the classic dichotomy in visual processing that associates object shape processing with ventral structures such as AIT but places spatial processing in a separate anatomical stream projecting to dorsal structures.

## Introduction

Visual processing is commonly divided into dorsal and ventral streams. The dorsal stream is associated with the representation of spatial relations connected to visuomotor control, while the ventral stream is involved in encoding shape and color information required for object identification and discrimination [1], [2]. Nevertheless, there is a great deal of interaction between the two streams [3], and it is increasingly apparent that both spatial and shape information are available in each stream. Physiological studies show shape information at high levels in the dorsal stream of the macaque monkey [4]–[8]. Also, fMRI studies in monkeys [9] and humans [10] show widespread shape selectivity in dorsal structures. In this report, we describe experiments showing the converse, that extensive spatial information is present within the ventral stream, in area TE of anterior inferotemporal cortex (AIT).

Area TE, a late visual area in the ventral stream, contains neurons that show specificity for complex shapes [11]–[13], with major output to perirhinal cortex and then the hippocampal complex [14]–[16]. TE is believed to be of central importance for perceptual aspects of object recognition. Lesion studies indicate that its removal impairs object discrimination but not visual acuity [17]–[19].

A major issue in theories of object recognition is whether the spatial frame of reference is object-centered [20]–[22] or viewer-centered [23]–[25]. Single-cell and fMRI data from monkey inferotemporal cortex (IT) or its human analog provide support for both object- [10], [26]–[28] as well as viewer-centered viewpoints [29], [30].

Given multiple objects arranged in a scene, a third spatial frame is possible, one in which positions are referenced to a landmark within the scene. Some evidence supports a scene-based frame of reference in inferotemporal cortex [28], [31]. Object-based and scene-based spatial frames are included in the term *allocentric*, denoting a coordinate system external to the viewer (world-centered), while viewer-centered spatial frames are termed *egocentric*.

It is a matter of debate which spatial frame of reference occurs in inferotemporal cortex. Clearly, however, some frame of reference must exist, and if the reference frame is not retinocentric, a coordinate transformation must occur. Spatial coordinate transforms have been extensively studied in posterior parietal cortex in the context of mechanisms for visuomotor control [32]–[34]. We suggest that coordinate transforms used to explain dorsal stream functionality can also be useful for understanding object representations in inferotemporal cortex.

**Figure 1:**
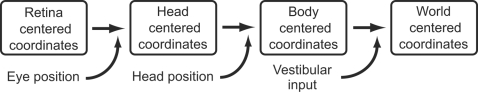
Diagram showing how postural inputs to inferotemporal cortex can be used to transform the spatial coordinate system of object representations. Although different inputs are shown serially, they could equally well occur in parallel at a single processing stage.

Neural computation of spatial coordinate transforms can be effected through *gain fields* [35]–[40]. In a gain field, visual responses of retinotopically-organized neurons are modulated by postural information, such as eye or neck position. This modulation does not affect the retinotopic organization of neurons nor their stimulus selectivity, but simply changes their response magnitude by a gain factor. Adding eye position modulation to retinotopic responses allows transformation from a retinocentric to a head-centered coordinate system (Figure 1). If head position modulation is also present, a body-centered (axial) reference frame is possible. Adding vestibular modulation transforms an egocentric to an allocentric representation.

Stimulus retinal position is a first requirement for implementing a gain field. Convincing evidence exists that retinotopic information is retained by inferotemporal neurons [41]–[43]. As part of this study, we will show that retinal position information is available in inferotemporal responses under our experimental conditions.

A second requirement is that retinotopic responses can be modulated by postural information. In this study, we specifically examined eye position modulation in inferotemporal cortex. Eye position effects are widely observed in dorsal stream structures [32], [44], but less data are available for the ventral stream. Eye position modulation has been found in macaque V4 [45], [46], and there is one previous report of eye position modulation in macaque inferotemporal cortex using much larger gaze angle shifts than we used [47]. Also, human fMRI studies have shown eye position modulation in the collateral sulcus [48], as well as evidence for a head-centered coordinate system rather than a retinocentric one in lateral occipital cortex [49].

Although we support the idea that dorsal and ventral visual pathways have very different goals, we argue that each pathway incorporates both spatial and object processing in implementing those different goals. Hence, it follows that there should not be a rigid anatomical separation between spatial and object processing. We test here whether a high-level ventral stream area shows retinal and eye position spatial sensitivities, and present robust evidence for spatial functionality within the ventral pathway.

## Materials and Methods

### Physiological preparation

Two male macaque monkeys (*Macaca mulatta*, 10 kg; *Macaca nemestrina*, 8 kg) were trained with two behavioral tasks. Before recording, a chamber centered 18 mm anterior to the ear bars and 10–12 mm lateral to midline was mounted on the skull, over the left cerebral hemisphere. All procedures were conducted in accordance with NIH Guidelines, reviewed and approved by the U. of Texas-Houston Animal Welfare Committee.

### Data collection

All encountered AIT cells that could be stably isolated were recorded from extracellularly, using either platinum-iridium or tungsten microelectrodes (1–2 MΩ, Microprobe). The receptive field of each cell was qualitatively mapped, typically with a probe stimulus presented at eight polar angle positions in the visual field with eccentricities between 1.5° and 12.0°. Shape sensitivity was then assessed using eight shapes presented at the most responsive location.

### Eye position monitoring and calibration

Eye position was monitored using a standard scleral eye coil. It was calibrated to screen coordinates by sampling fixation position (averaged across a 50 ms period) after the animal had stably fixated for 350 ms at each target location (8 or 3 depending on task) as well as at central fixation. Any offsets from those positions could be converted to visual angle by interpolating across the calibration points. During each trial, animals had to maintain eye position within a small window (0.5°, half-width) around a central fixation point. Then, after presentation of the saccade target, the animals were required to make a single saccade to target with landing position within a small window (up to 0.6° half-width, across all recorded neurons) centered on the target position. Trials were aborted and the animals received no reward if their eye position left the central fixation window before target presentation, or if their landing position did not reach the correct target window.

### Visual stimuli and behavioral tasks

**Figure 2:**
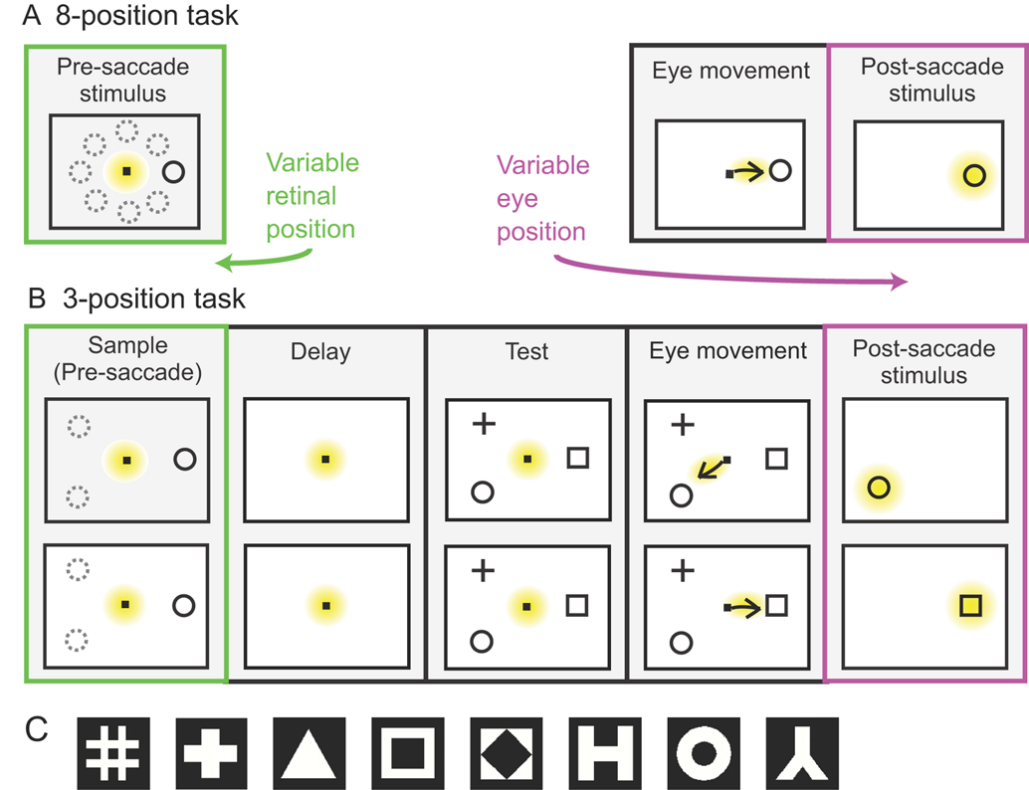
Summary of the experimental design. Early in the trial the monkey was centrally fixated, and stimuli appeared at different peripheral retinal positions. The monkey then made a saccade to the stimulus. At this point the stimulus was fixated, but the eye was at different positions (gaze angles). A. 8-position task: the monkey made a saccade to the stimulus immediately following its appearance. Stimuli appeared at 8 possible locations (dashed circles). B. 3-position task: the monkey performed a delayed match to sample task, either match-to-shape (top) or match-to-location (bottom). Stimuli appeared in 3 possible locations (dashed circles). For present purposes, we were interested only in the sample period (retinal position effects) or the post-saccade period (eye position effects). c. Set of shapes used.

Stimuli were displayed on a 20-inch, 75-Hz CRT monitor with a resolution of 1152×864 pixels, placed 65 cm in front of the animal (36°×27° visual angle). Beyond the monitor was a featureless black screen (54°×40° visual angle). Experiments were conducted in a darkened room. Shape stimuli were selected from a set of eight simple geometric forms, all consisting of white and black pixels in equal numbers (Figure 2C). The stimulus size ranged from 0.65° to 2.00° (mean: 0.8°), increasing with eccentricity.

We examined AIT neurons for eye position effects using two widely used behavioral paradigms: a delayed match to sample task and a prosaccade task. Since the emphasis here is on spatial position rather than cognitive effects, we refer to them as the 3-position task and 8-position task respectively (Figure 2A,B). Making eye movements in the context of behavioral tasks may offer a more ecologically valid context for examining eye position effects than the common practice of flashing stimuli to eyes statically held at various oblique angles. More importantly, this design allowed examination of the temporal development of eye position effects as the eyes moved to a new location. Eye position effects were measured with the stimulus at fixation, but with the eyes at different gaze angles. This experimental design is appropriate for AIT neurons, as their receptive fields have maximal responses close to fixation [42], in contrast to parietal neurons, for example, which frequently have foveal-sparing responses [50].

### i. 8-Position task

For each cell, preliminary testing determined the most effective stimulus shape (among the eight possible), and that shape was used in all trials. There were eight possible stimulus positions. The polar angles of the eight positions covered a full 360° in approximate 45° increments. There was a median of 12 trials per location (min: 5; max: 12). Eccentricities for all positions were constant for a given cell, with eccentricities for different cells ranging from 2.1°–6.9° (mean: 3.9°). All 8 positions were used once in random order to form a block, before being used again in the next block.

Each trial began with the presentation of a fixation point at the display center (Figure 2A). A target stimulus then appeared at one of eight peripheral locations. The animal was required to make an immediate saccade to the target to obtain a liquid reward. When the eye position entered the target window, the fixation point was extinguished and the target persisted on the screen for 400 ms. The monkeys' performance on this task averaged 92% correct.

### ii. 3-position task

Preliminary testing of each cell determined a highly responsive position in the visual field. That position, plus two others ±120° from the polar angle of the preferred position (all at the same eccentricity) gave the three stimulus locations tested. There was a median of 64 trials per location (min: 28; max: 72). Eccentricities for different cells ranged from 2.0°–10.0° (mean: 4.1°). Responses at each position were examined with three stimulus shapes. The three shapes were selected based on initial screening that identified the two most effective shapes in our set, plus the least effective shape.

For each trial the eyes were initially centrally fixated on a spot (Figure 2B). A sample stimulus shape, randomly selected from three possibilities, appeared at one of three peripheral locations for 340–410 ms. Following this sample presentation, there was a random delay period of 600–2700 ms, during which the screen was blank except for the fixation spot. After the delay, three target stimuli appeared, each with a different shape and location. The monkey was required to make an immediate saccade to the stimulus that matched either the shape (shape-match subtask) or the location (location-match subtask) of the sample stimulus, the subtask having been indicated by a cue at the beginning of the trial. When the eye position reached the correct target window, all other stimuli on the screen were extinguished. The target stimulus stayed on for 400 ms after the saccade reached its position.

All combinations of the three shapes and three positions presented were used once in random order to form a block, before being used again in the next block. In some cases the two subtasks were presented in separate alternating blocks (9 trials/block) and in other cases they were intermixed within one large block (18 trials/block). Performance on this task averaged 81%.

### Data analysis

**Figure 3:**
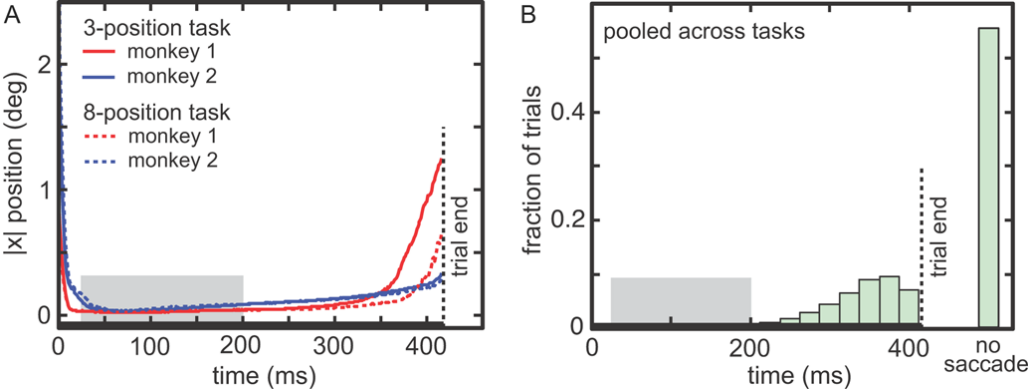
A. Average eye trace (x-position) during target fixation. Median absolute eye position across all trials calculated at each time point. Absolute value of eye position was used to pool saccades in different directions. Zero time marks when eye left the central fixation window. Downward portion of the curve on left represents target arrival saccade from central fixation. Flat middle portion is target fixation period. The average fixation position was normalized to zero on a trial-by-trial basis. Upward portion of curve on right shows target departure saccade to a random location, following successful completion of the task. Gray box indicates time period used for ANOVA. B. Distribution of saccade times before target offset, pooling data for the two tasks.

We used eye trace records to select trials for analysis that excluded saccades (Figure 3B). The criteria for a saccade were an eye velocity greater than 30 deg/sec and a change in position greater than 0.5°.

Data from the 3-position task were analyzed using a three-way ANOVA with the factors being position (retinal or eye), stimulus shape, and subtask. For data from the 8-position task a one-way ANOVA with factor position (retinal or eye) was used. Significance level of ANOVA was p<0.05.

In addition to this broad-period (175 ms) ANOVA, we examined the time course of eye position effects with ANOVA over a short duration sliding time window. Only cells with a significant eye position effect under the broad-period ANOVA were studied. The sliding window width was 25 ms for the 3-position task, 35 ms for the 8-position task (wider in the latter case to compensate for a smaller sample size), incremented in 2 ms steps. We included only trials in which the target remained fixated until trial end. Trials from all significant cells were combined into one pool, rather than performing the analysis on a cell-by-cell basis in order to increase statistical power and allow narrower time windows, improving the temporal resolution of the results.

For each cell, we calculated a position selectivity index defined as *SI* = (*r*_max_−*r*_min_)/(*r*_max_+*r*_min_) where *r*_max_ and *r*_min_ were maximum and minimum responses across all positions. This formula was used for both retinal position and eye position selectivity. Calculations of position SI were based on the most effective shape. Standard errors of SI were estimated by bootstrap resampling of trials from best and worst positions.

## Results

Data were collected from 143 cells in AIT, almost all in area TE. These cells fell along the lower bank of the superior temporal sulcus (STS) and the convexity of the middle temporal gyrus, within the anterior-posterior range of A14–A22. A few perirhinal cells were included at the extreme anterior portion of that range. A diagram of the histology has previously been presented [4]. All 143 cells were tested with the 3-position task. In addition, 80 of the 143 cells were tested with the 8-position task. The average latency for these cells was 92 ms, measured as time to half-height of peak response in the pooled peristimulus time histogram.

### i. Eye tracking data

Within each trial, different time epochs were selected for analysis based on eye position (Figure 2). For the retinal position effect, we were interested in the period when the monkey's gaze was centrally positioned on the fixation spot and the stimulus appeared eccentrically. For the eye position effect, we analyzed the period when the monkey was eccentrically fixated on a target. Those periods were based on examination of the eye tracking traces.

Figure 3A plots the median eye trace for the time epoch when the monkeys were fixating the eccentric target stimulus. The flat horizontal portion of the curve in the center indicates the target fixation period. Figure 3B shows the distribution of times for the target departure saccade, with similar data from the two tasks pooled. The time of the departure saccade from target was greater than 200 ms in 98% of trials and the eyes remained fixated on the target for over 400 ms in 56% of trials. Based on these eye trace data, we selected, for purposes of data analysis, the period 25–200 ms (gray period in Figure 3A,B) as the target fixation period for the eye position effect for both the 3-position and 8-position task. This behavioral time window was shifted by average neural latency to select the spike train window used for analysis. Changing the endpoint of this window by ±50 ms did not significantly alter any of the reported findings.

In measuring the retinal position effect, there was no eye movement immediately after the sample period of the 3-position task. In that case the spike train time window for data analysis was simply stimulus duration (mean: 370 ms) shifted by neural latency. For the 8-position task, the spike train window went from target onset in the periphery until the monkey left the central fixation window (mean: 181 ms), again shifted by neural latency.

### ii. Retinal position effect

We examined the effect of placing the same stimulus shape at different retinal positions while the monkey maintained central fixation. ANOVA results are shown in Tables 1 and 2. A majority of cells had responses that significantly depended on the retinal position of the stimulus– 62.2% during the 3-position task and 66.3% during the 8-position task. There was a population bias towards the most responsive area occurring in the contralateral hemifield, consistent with previous reports [42], [51].

**Table 1:** Retina position effects for 3-position task (ANOVA, p<0.05).

Main effect	Significant units
Position	62.2% (89/143)
Shape	83.2% (119/143)
Subtask	28.7% (41/143)
**2-way interactions**	
Position*shape	39.2% (56/143)
Position*subtask	7.0% (10/143)
Shape*subtask	12.6% (18/143)

**Table 2:** Retina position effects for 8-position task (ANOVA, p<0.05).

Main effect	Significant units
Position	66.3% (53/80)

**Figure 4:**
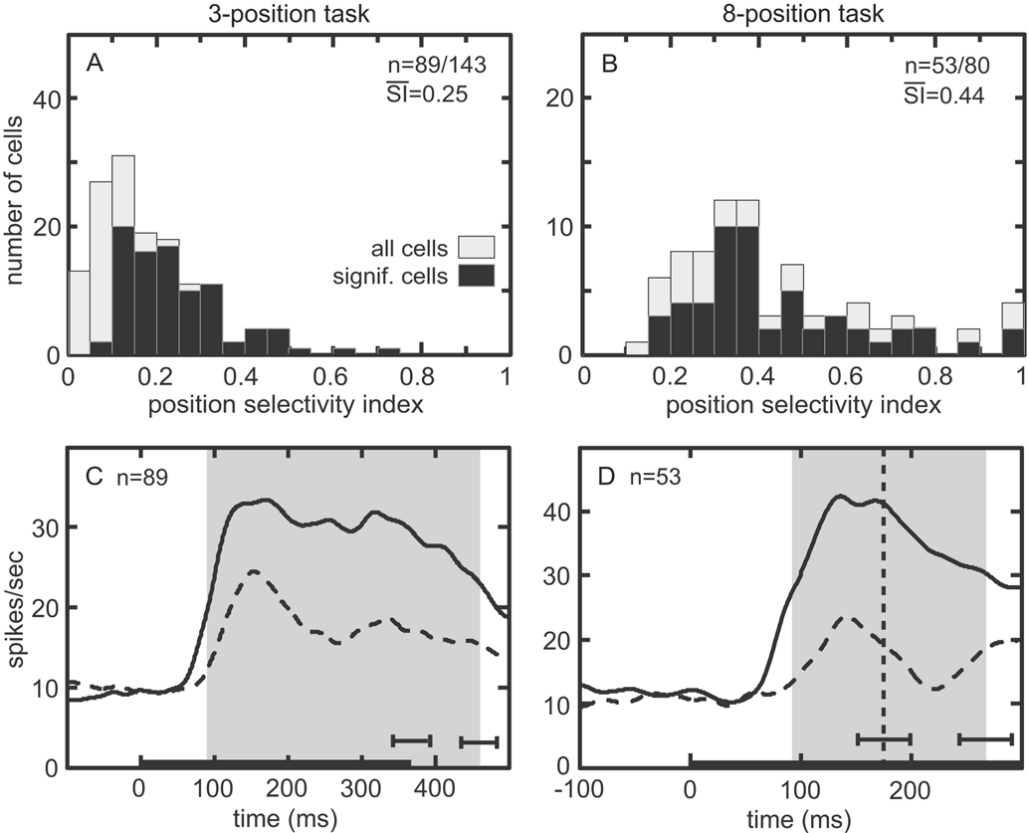
Retinal position effect showing stimulus response changes as a function of retinal position. A. Distribution of position selectivity index (SI) for 3-position task. Larger values indicate greater selectivity. Mean SI for significant cells is indicated. B. Distribution of position selectivity index for 8-position task. C. Time course of responses at best and worst locations for 3-position task, averaged over all significant cells. Data included from the most effective shape for each cell. Trials were aligned on stimulus onset (zero time). Gray zone indicates stimulus period shifted by neural latency, the time epoch used for ANOVA. Horizontal error bars shows standard deviation of stimulus-off time and of stimulus-off time shifted by neural latency. Curves smoothed using a 10 ms Gaussian kernel. D. Time course of responses at best and worst positions for 8-position task. Gray zone indicates the period from stimulus start until saccade onset shifted by neural latency, used for ANOVA. Horizontal error bars indicate standard deviation of saccade onset time and of saccade onset time shifted by neural latency.

The distributions of the retinal position selectivity indices (SIs) for both tasks are shown in Figure 4A,B. For cells with a significant retinal position effect, the mean SI for the 3-position task was 0.25±0.05 with the SE of the mean estimated by bootstrap. That corresponds to a 67% increase between best and worst positions. For the 8-position task, mean SI was 0.44±0.06, corresponding to a 157% increase.

Time courses of responses to stimuli at best and worst retinal positions in the 3-position task are shown in Figure 4C, averaged over all cells showing a significant retinal position effect in the ANOVA, but including data from only the most effective shape. Responses at the best and worst positions in the 8-position task are shown in Figure 4D. In addition to position selectivity, 83.2% of the cells in the 3-position task were significantly selective for shape (Table 1). We examined to what extent retinal position affected the rank order of responses to different shapes in cells showing significant main effects for both retinal position and shape (58.7%, 84/143 cells). Counting instances where the first and second most effective shapes were not significantly different at the p = 0.05 level as “ties” for ranking purposes, the same shape was the most effective stimulus at all retinal locations in 97.6% (82/84) of the cells, and the Spearman rank order correlation coefficient was 0.79 (average correlation over all possible pairs of retinal positions). Without adjustment for “ties”, the same shape was the most effective stimulus at all retinal locations in 77.4% (65/84) of the cells, and the Spearman coefficient was 0.78. Chance performance would be 11% of cells having the same most effective stimulus at all retinal positions.

### iii. Eye position effect

**Figure 5:**
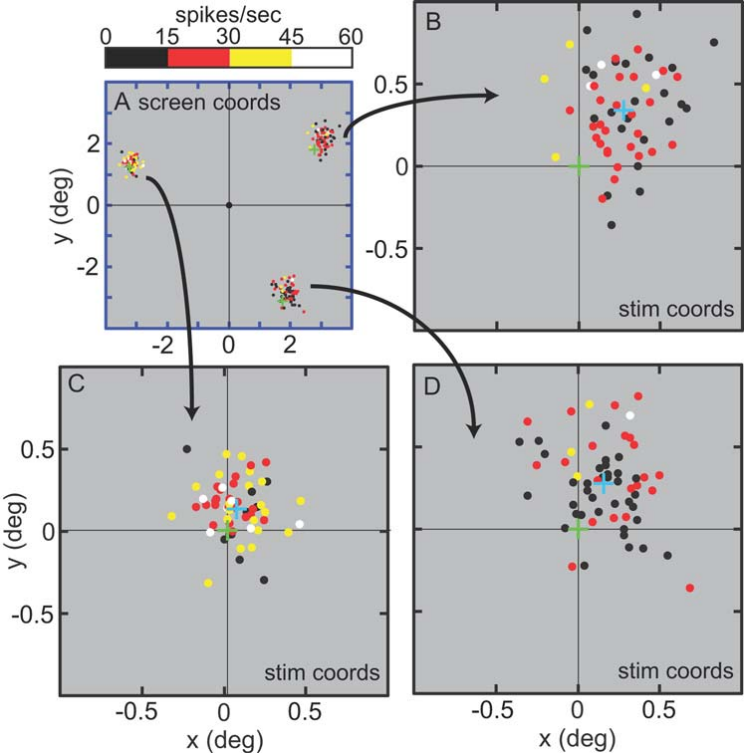
Saccade endpoint scatter for example cell in the 3-position task. Each dot represents the endpoint for a single trial, and dot color indicates firing rate for that trial. A. Global view showing endpoints for all three stimulus locations. Green cross indicates stimulus center at each location. Coordinates are relative to central fixation point. B,C,D. Magnified views of individual stimulus locations. Green cross indicates stimulus center, and blue cross indicates average saccade landing point. Coordinates are relative to stimulus center.

We examined the effect of gaze angle on responses to stimulus shape. Figure 5 shows responses at different eye positions for an example cell. Each dot indicates the saccade landing point for one trial and the color of the dot indicates firing rate. Saccade landing point was defined as average eye position during the stimulus fixation period (25–200 ms) following saccade onset and correct target acquisition (gray period in Figure 3A,B). The figure suggests that firing rate of this cell changed for the three eye positions, with the highest rate for a gaze angle toward the upper left (magnified in Figure 5C). ANOVA results for the recorded population are given in Tables 3 and 4. Almost half of the inferotemporal cells tested had responses that significantly depended on eye position: 45.5% during the 3-position task and 41.3% during the 8-position task.

**Table 3:** Eye position effects for 3-position task (ANOVA, p<0.05).

Main effect	Significant units
Position	45.5% (65/143)
Shape	79.0% (113/143)
Subtask	19.6% (28/143)
**2-way interactions**	
Position*shape	26.6% (38/143)
Position*subtask	7.7% (11/143)
Shape*subtask	12.6% (18/143)

**Table 4:** Eye position effects for 8-position task (ANOVA, p<0.05).

Main effect	Significant units
Position	41.3% (33/80)

**Figure 6 pone-0003492-g006:**
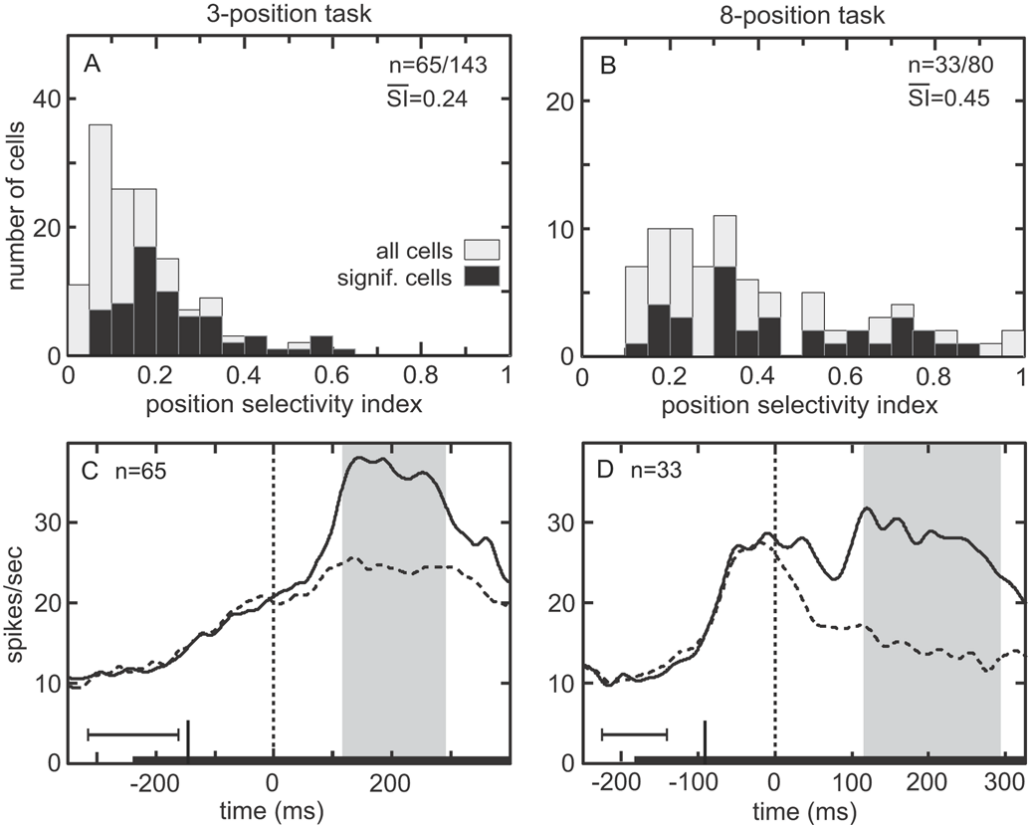
Eye position effect showing stimulus response changes as a function of gaze angle while stimulus remained foveally fixated. A. Distribution of position SI for 3-position task. B. Distribution of position SI for 8-position task. C. Time course of responses at best and worst eye positions in the 3-position task, averaged over all significant cells. Data included from most effective shape for each cell. Trials aligned on saccade (zero time). Gray zone indicates target fixation period, shifted by neural latency, the time epoch used for ANOVA. Horizontal error bar shows standard deviation of stimulus onset time. Long vertical tick mark indicates target onset shifted by neural latency. Curves smoothed using a 10 ms Gaussian kernel. D. Average time course at best and worst eye positions for 8-position task. Conventions are the same as in Figure 6C.

The distributions of the eye position selectivity indices (SIs) for both tasks are shown in Figure 6A,B. For cells with a significant eye position effect, the mean SI for the 3-position task was 0.24±0.07 with the SE of the mean estimated by bootstrap. That corresponds to a 63% increase between best and worst positions. For the 8-position task, mean SI was 0.45±0.08, corresponding to a 164% increase.

All eighty cells tested on the 8-position task were also tested on the 3-position task. Of these, 48.8% (39/80) showed a significant eye position effect during the 3-position task and 41.3% (33/80) during the 8-position task, while 25.0% (20/80) showed a significant effect for both tasks. Among cells significant for both tasks, the SI was greater for the 8-position task by an average of 0.21.

Cells with significant eye position selectivity were examined for a population bias for a particular best gaze angle using a Kuiper test on data from the 8-position task. Probability distributions of best positions versus non-best positions were compared, with position parameterized as the polar angle of eye angle. We found no significant difference in the two distributions (p>0.85), indicating no population bias for a preferred gaze angle.

From trial to trial there was scatter in saccade landing points on the target (e.g. Figure 5). For cells showing a significant eye position effect, the circular error probability (CEP) of the landing points (i.e., the radius of a circle containing 50% of the landing points) was 0.21°. The distance of the average landing spot from the stimulus center was 0.20°. For each cell, the average landing spot for each eye position was close to the same point on the target. Across all cells with significant eye position effects, landing positions were on average 0.11° from the grand average landing spot (pooled over all locations in the task). Within a single trial, the CEP of eye position during target fixation was 0.04°. Measurements of human saccades to extended targets show that the average landing points are close to the stimulus center, with accuracy and precision similar to that reported here [52], [53].

The time courses of responses to stimuli at best and worst eye positions in the 3-position task are shown in Figure 6C, averaged over all cells showing a significant eye position effect in the ANOVA, but including data from only the most effective shape. Figure 6D shows results for the 8-position task, again showing a large gaze angle effect. In addition to eye position selectivity, 79.0% of the cells in the 3-position task were significantly selective for shape (Table 3). We examined to what extent eye position affected the rank order of responses to different shapes for all cells showing significant main effects for both eye position and shape (40.6%, 58/143 cells). Allowing for “ties” in rank (i.e., when first and second best shapes were not significantly different), the same shape was the most effective stimulus at all eye positions in 91.4% (53/58) of the cells, and the Spearman rank order correlation coefficient was 0.71 (average correlation over all possible pairs of eye positions). Without adjustment for “ties”, those numbers were 67.2% (39/58) and 0.73. Chance performance would be 11% of cells having the same most effective shape at all eye positions.

**Figure 7 pone-0003492-g007:**
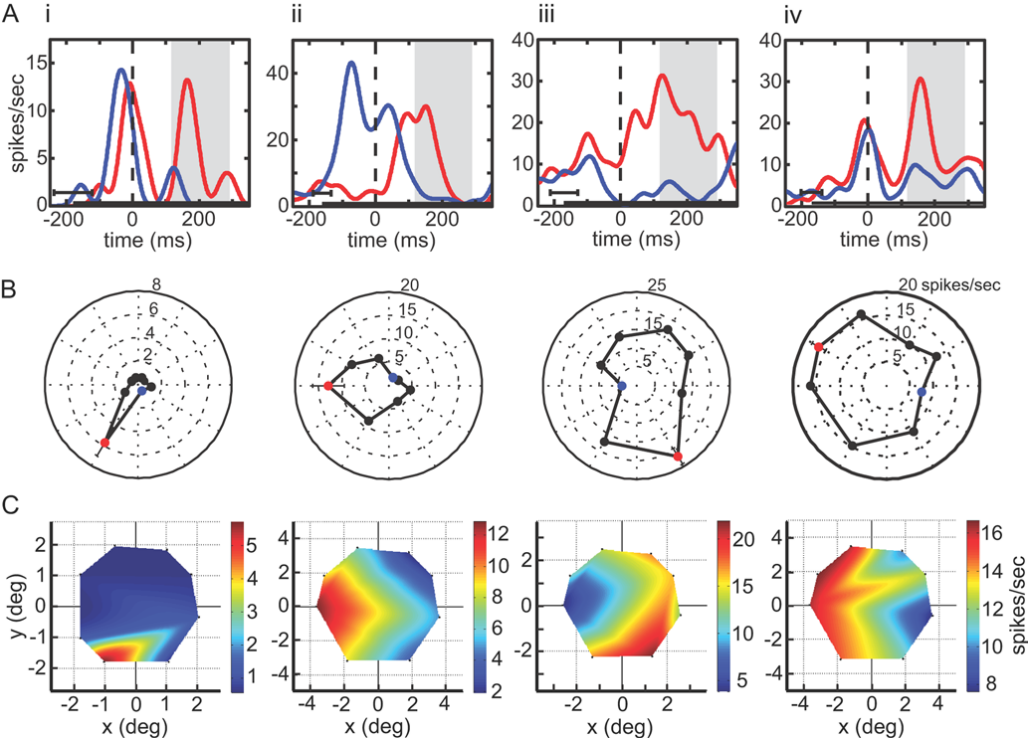
Four example cells showing eye position effects during 8-position task. A. Time course of responses at best (red) and worst (blue) eye positions. Gray period indicates target fixation period shifted by neural latency. Curves smoothed using a 20 ms Gaussian kernel. B. Polar plots showing eye position effect. Radial dimension indicates firing rate, and angle dimension represents polar angle of stimulus position. Blue and red dots indicate positions producing lowest and highest responses. C. Interpolated response surface, showing firing rate as a function of eye position. Surface was generated by cubic spline interpolation through the eight data points (vertices of colored region).

Data from four example cells recorded during the 8-position task are shown in Figure 7. Each column gives three perspectives of the data from one cell. In the top panel are the time courses of responses for the best (red) and worst (blue) eye positions over the eight possible positions. In some cases two peaks of activity are apparent (see e.g. Figure 7A, neuron i), before and after the saccade (vertical dashed line). The first peak is the stimulus response during the pre-saccade period (Figure 2A, green box), when the eye is centrally fixated and the stimulus appears peripherally. The second peak occurs after the eye position changes and the eye is fixated on the stimulus (Figure 2A, magenta box). A polar plot in the middle panel of Figure 7 shows how the response magnitude changes at different eye positions. In the bottom panel, a response surface (firing rate as a function of eye position) has been interpolated between the data points located at the eight vertices, using cubic spline interpolation.

The ANOVA results presented in Tables 3 and 4 used a broad, 175 ms time window. Once a set of cells showing a significant eye position effect had been identified using this broad-period analysis, we reexamined their responses at a finer temporal resolution using an ANOVA with a narrow (25–35 ms) sliding time window (2 ms increments). This allowed us to observe the development of the eye position effect over time.

**Figure 8:**
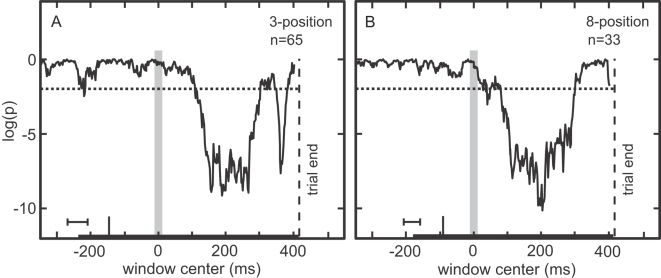
Development over time of eye position effect following saccade to target, for both (A) 3-position task and (B) 8-position task. Data within a sliding time window were examined using ANOVA. Zero time marks departure of eye from central fixation window, and vertical gray bar is an estimate of saccade duration. Thick horizontal axis bar indicates target duration, and long vertical tick is target onset shifted by average neural latency. Vertical axis is logarithm of the significance level of eye position effect. Horizontal dotted line is p = 0.01 level of significance.

As illustrated in Figure 8, the sliding window analysis shows a large, highly significant eye position effect developing after the eye movement to target. The time at which the ANOVA curve crossed the p = 0.01 criterion (eye position effect latency) was 107 ms following saccade onset for the 3-position task and 79 ms for the 8-position task, with a smaller brief deflection occurring earlier at 35 ms.

The eye position effect was not due to large receptive fields being affected by changing stimulus conditions beyond the edge of our display apparatus. Receptive field diameters of 32° or less would have remained entirely within the monitor and surrounding screen. Recent estimates of average AIT receptive field diameters are in the 10°–16° range [42], [54], [55], substantially smaller than earlier reports [56], [57], with the added observation that receptive field sizes may shrink to even less for small stimulus sizes such as ours [42]. Furthermore, we know the responses of these cells to peripheral stimulation by examining their activity prior to the saccade to target, when the target was in the periphery. For cells with significant eye position effects, peripheral stimulation using a high contrast shape specifically selected as effective for each cell and located at a small eccentricity (average 4°) failed to produce population-averaged responses that mimicked an eye position effect (see pre-saccade period of Figure 6C,D).

## Discussion

This study demonstrates clearly that spatial information is available in the visual responses of area TE of AIT, in the form of modulations arising from changes in stimulus retinal position as well as changes in eye position (gaze angle).

With respect to retinal position, about two-thirds of AIT neurons were sensitive to modest shifts in the retinal stimulus location (Figure 4). While from one perspective this retinotopic modulation is simply a manifestation of the existence of a receptive field, from another perspective it indicates that inferotemporal cortex retains information about the spatial position of objects in retinocentric coordinates. This is contrary to the widespread assumption of translational invariance of inferotemporal cortex responses in computational models of object recognition. These observations of retinal position selectivity concur with previous findings [41]–[43], and suggest that retinotopic spatial modulation in inferotemporal cortex is qualitatively similar to that found in parietal cortex [50].

Regarding eye position effects, we demonstrate that the visual responses of nearly half of AIT neurons vary strongly with small shifts in gaze angle (Figures 5–8). These robust findings in AIT are similar to previous reports of eye position effects in higher dorsal stream areas, such as 7a and LIP [58]. Possible sources of eye position information can include proprioceptive feedback from ocular muscles and motor efference copy [59], [60]. Our data showed eye position effects starting tens of milliseconds after the saccade to target (Figure 8), a timing most consistent with a proprioceptive source with afferent delay [60] though it cannot exclude motor efference copy.

The eye position effect cannot be due to receptive field remapping. Predictive remapping effects occur immediately before eye movements [61], [62]. Our effects begin after the eye movement to target, and also more than 300 ms before an eye movement that might occur after the end of the trial. Thus, the time course of our eye position effect is inconsistent with remapping. Moreover, it is unlikely the effects reflect a motor planning signal in inferotemporal cortex. The effects diminish toward the end of the trial (Figure 8), the opposite of what would be expected if it were due to motor planning for a future saccade. As these cells had phasic responses, the drop in eye position response modulation at the end may have been related to the drop in overall response levels.

There is one previous report of eye position modulation in inferotemporal cortex for very large gaze angle shifts (10–40°) using large fixation windows of up to 5° [47]. However, recent reports of inferotemporal cells, as well as the current study, suggest that the size of those windows could allow for significant differences in response due to retinal position [41], [42]. Further, as small saccades occur more frequently, we were interested in examining the effects of smaller eye position shifts under highly controlled conditions.

The modulation of visual responses by eye position, widely observed in parietal cortex, has been interpreted there as a mechanism for performing a coordinate transform from a retina-centered to a head-centered frame of reference. A similar interpretation can be given to the eye position modulations we observe in inferotemporal cortex, providing a means by which a non-retinocentric spatial frame of reference can be computed in inferotemporal cortex based on postural inputs (Figure 1). This integration of sensory and postural inputs at late stages of visual object processing, as well as in other visually responsive areas, reinforces the view of vision as an active, embodied process rather than a passive representational process [63].

Modulation of visual responses by eye position has been observed in widely dispersed areas. These include late stages of the dorsal stream (7a and LIP: [34], [58], [64]), frontal areas (premotor and supplementary eye fields: [65], [66]), early stages of visual processing (striate cortex: [67], [68]), thalamus (pulvinar: [69]), and in V4, a mid-level ventral stream cortical area [45]. The widespread occurrence of such similar modulations led to the speculation [45] that the same spatial frame of reference could be used simultaneously across different cortical areas and streams of processing. However, we would argue that is not the case. Neurons in each structure can receive postural information from more than one source at the same time (eye position, neck position, etc.; Figure 1), and the frame of reference would then be dependent on the combined pattern of such postural inputs. Therefore, despite observations of similar eye position modulations in dorsal and ventral stream structures, it does not follow that both streams are likely to encode space in the same manner. We have previously demonstrated such a difference in encoding between the streams with respect to object shape properties [4], in which population representations of shapes within the ventral stream are both more distinctly delineated and better categorized with respect to perceptual similarity than in the dorsal stream.

A notable aspect of spatial reference frames encoded by postural modulations of visual activity is that they are implicitly represented in the population response rather than explicitly in the responses of individual neurons [32], [36], [37]. For example, although gain fields for eye position can perform a coordinate transform from retinocentric to head-centered coordinates, the resulting receptive fields of individual neurons are not tied to particular locations in head-centered coordinates but remain retinocentric (although modulated by gaze angle). Information about stimulus location in head-centered coordinates is distributed across such a population of modulated cells. However, by examining the visual activity of inferotemporal neurons for easily measurable postural modulations, as we do here, we may infer the presence of such population-coded spatial representations that are otherwise not directly apparent at the single neuron level.

Our finding that both retinotopic and gaze angle spatial information are embedded within neural responses at a high level of the ventral stream parallels observations of shape selectivity within the dorsal stream [5]. The distinction between ventral and dorsal processing, therefore, is not adequately captured by a dichotomy between object and spatial representations. Rather, the difference lies in how both object (shape, color) and spatial information are combined in different ways in the two streams to achieve different goals [70]. Furthermore, we propose that the specific nature of object and spatial information is different in the two streams (see, e.g., [4] for differences in encoding of object shape). The ventral stream may be engaged in constructing a model of the world within an allocentric reference frame, in which object identities and spatial relationships are encoded for purposes of relatively abstract, goal-directed planning. Such a representational format may also serve well as input to long-term memory. An allocentric mode of spatial processing in ventral pathways is consistent with the pattern of behavioral deficits observed in a patient with ventral lesions [71]. The dorsal pathway, on the other hand, may specialize in functions requiring an egocentric frame of reference, including most cases of real-time visuo-motor control.

In conclusion, we report the presence of robust spatial information in many neurons in AIT, a high level visual area in the ventral stream important for object recognition and a major conduit of visual signals to memory-related structures in the hippocampal complex. Our data show that spatial modulation of visual responses is not restricted to visual areas associated with the dorsal stream. The finding that inferotemporal cortex contains sufficient spatial information to compute a non-retinocentric frame of reference based on postural inputs alters, in a fundamental way, the current view of the nature of visual processing in the ventral stream and provides strong grounds for revising the classic segregation of shape versus spatial processing in ventral and dorsal cortical visual streams.
